# Desaturase-dependent secretory functions of hepatocyte-like cells control systemic lipid metabolism during starvation in *Drosophila*

**DOI:** 10.21203/rs.3.rs-5566817/v1

**Published:** 2024-12-11

**Authors:** Jiayi Li, Kerui Huang, Indira Dibra, Ying Liu, Norbert Perrimon, Matias Simons

**Affiliations:** 1 Nephrogenetics unit, Institute of Human Genetics, University Hospital Heidelberg, Heidelberg, Germany; 2 Molecular Medicine Partnership Unit (MMPU), University of Heidelberg and European Molecular Biology Laboratory (EMBL), Heidelberg, Germany; 3 Department of Genetics, Blavatnik Institute, Harvard Medical School, Boston, MA, USA.; 4 Howard Hughes Medical Institute, Harvard Medical School, Boston, MA, USA.

## Abstract

Similar to the mammalian hepatocytes, *Drosophila* oenocytes accumulate fat during fasting, but it is unclear how they communicate with the fat body, the major lipid source. Using a modified protocol for prolonged starvation, we show that knockdown (KD) of the sole delta 9 desaturase, Desat1 (SCD in mammals), specifically in oenocytes leads to more saturated lipids in the hemolymph and reduced triacylglycerol (TAG) storage in the fat body. Additionally, oenocytes with *Desat1* KD exhibited an accumulation of lipoproteins and actin filaments at the cortex, which decreased lipoproteins in the hemolymph. We further show that ImpL2 (IGFBP7 in mammals) is secreted from oenocytes during starvation in a *Desat1*-dependent manner. Flies with oenocyte-specific KD and overexpression of ImpL2 exhibited higher and lower sensitivity to starvation as well as lower and higher levels of TAG, respectively. Intriguingly, the depolymerization of cortical actin in the oenocytes decreased lipoprotein sequestration and alleviated the secretion defect of *Impl2* in *Desat1* KD cells, leading to rescued TAG levels and starvation sensitivity. Overall, this study highlights the central role of oenocytes in systemic lipid metabolism in *Drosophila* as well as the importance of Desat1 in maintaining the proper functioning of oenocytes during periods of starvation.

## Introduction

Adaptation to changing environmental conditions is essential for survival. When animals adapt to nutrient shortage, intra-organ responses as well as inter-organ crosstalk mechanisms need to be mounted to coordinate whole-body energy homeostasis. A central organ in this process is the liver. In early stages of fasting, the glycogen stores of the liver are key for maintaining blood glucose levels. Upon prolonged fasting, there is a gradual transition in the body’s energy source from carbohydrates to lipids reflected by the accumulation of lipids in the liver that are provided from lipolysis occurring in the adipose tissue. While the increase in circulating free fatty acids stimulates the production of ketone bodies in the liver, starvation also leads to major changes in the composition of circulating lipoproteins. In humans, for example, a shift towards more unsaturated triacylglycerol (TAG) and sterol esters in the serum has been observed^1^. All these processes are orchestrated by an array of hormones and other bioactive molecules such as hepatokines, adipokines or myokines^2^.

*Drosophila melanogaster* has emerged as a major model system for the study of metabolic organ cross-talk^3^. In *Drosophila*, the fat body (FB) has been considered analogous to the human liver due to its roles in lipoprotein production and glycogen metabolism. However, its major function is to store fat similar to the adipose tissue. Oenocytes, on the other hand, were primarily associated with cuticular hydrocarbon and pheromone synthesis, until Gutierrez et al. demonstrated that oenocytes can accumulate lipids during periods of starvation similar to the liver^4,5^. Lipids accumulating in the oenocytes during fasting originate from the FB and are regulated by the insulin-like peptide, dILP6, which is secreted by the FB^6^. This behavior suggests a lipid exchange and crosstalk between the FB and oenocytes, analogous to the communication between adipose tissue and liver^7,8,9^.

Why oenocytes store lipids during fasting is not understood. While one reasonable assumption is that, also in flies, lipids are used for ketogenesis, lipids may also be modified and stored as TAG by the oenocytes before being released again into the circulation. Similar to humans, the modifications could involve lipid unsaturation. In this regard, it is interesting that Desat1 is highly expressed in oenocytes^4,10^. Desat1, the sole delta9 desaturase in *Drosophila* (corresponding to steaoryl-CoA desaturases (SCDs) in mammals)^11^, converts saturated into monounsaturated fatty acids and has previously been suggested to promote hydrocarbon formation in oenocytes^4^. However, as efficient TAG formation depends on SCDs in mammalian cells^12,13^, lipid modification by oenocyte Desat1 may also promote global TAG storage, which has been shown to be essential for survival under fasting conditions^14,15^

Like in mammals, important vehicles for lipids in the hemolymph, the fly’s circulatory system, are lipoproteins. The apoB-family lipoprotein, Lipophorin (Lpp), is synthesized and secreted by the FB and serves as the primary lipid carrier in the hemolymph. Under normal feeding conditions, Lpp transport to the midgut is crucial where it is loaded with sterols, medium-sized diacylglycerol (DAG) and phosphatidylethanolamine (PE) obtained by *de novo*–synthesis or from the diet. Lpp loading on the enterocyte surface depends on another FB-derived apoB-family lipoprotein, Lipid Transfer Particle (LTP), which enters the enterocytes via endocytosis while Lpp remains bound to the surface^16^. In the wing disc, on the other hand, the low-density lipoprotein (LDL) receptor orthologues LpR1/2 promote Lpp internalization, while also mediating non-endocytic lipid uptake mechanisms^17,18^. These examples illustrate the different modes of interactions of Lpp with its target cells. How lipoproteins mediate the transfer of lipids from the FB to oenocytes and vice versa in fasting conditions is unclear.

Here, we use *Drosophila* to highlight the role of oenocytes and Desat1 in the starvation response. We show that prolonged starvation causes alterations in the circulating lipidome and affects lipid storage and release of lipoproteins by oenocytes. Desat1 in oenocytes is essential for inducing these lipidomic changes by promoting lipid flux through the TAG compartment in oenocytes and by controlling lipid storage in the FB. The latter is facilitated by promoting the secretion of ImpL2 that controls FB lipolysis and promotes starvation resistance.

## Results

### Lipidomic shifts in *Drosophila* during nutrient restriction

**Figure 1:**
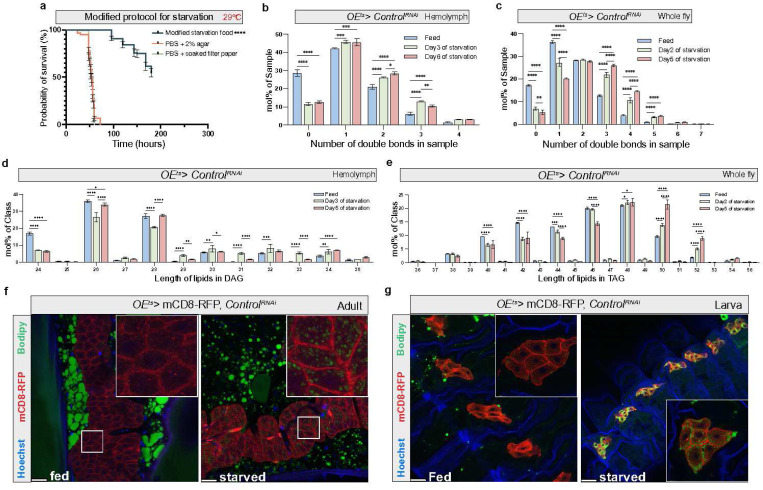
Lipidome shifts in adult Drosophila during starvation a, Starvation sensitivity assay using a modified protocol for long-term starvation experiments, n≈120 adult female flies. P value was calculated using Log-rank (Mantel-Cox) test. PBS, Phosphate Buffered Saline. b,c, The number of double bonds in hemolymph or whole fly samples at different days of starvation, n=3. Statistical tests: two-way ANOVA with Tukey’s multiple comparisons test. d,e, Length analysis of major lipids class from hemolymph or whole fly samples, n=3. Statistical tests: two-way ANOVA with Tukey’s multiple comparisons test. f,g, Lipid droplets (LD) were visualized with BODIPY (green) during modified starvation protocol in adult flies and during PBS starvation in larvae. Cell outlines were marked with mCD8-RFP (red). Scale bars, 20μm. *, P< 0.05; **, P<0.01; ***, P<0.001; ****, P<0.0001. Error bars indicate standard deviation

**Figure 2:**
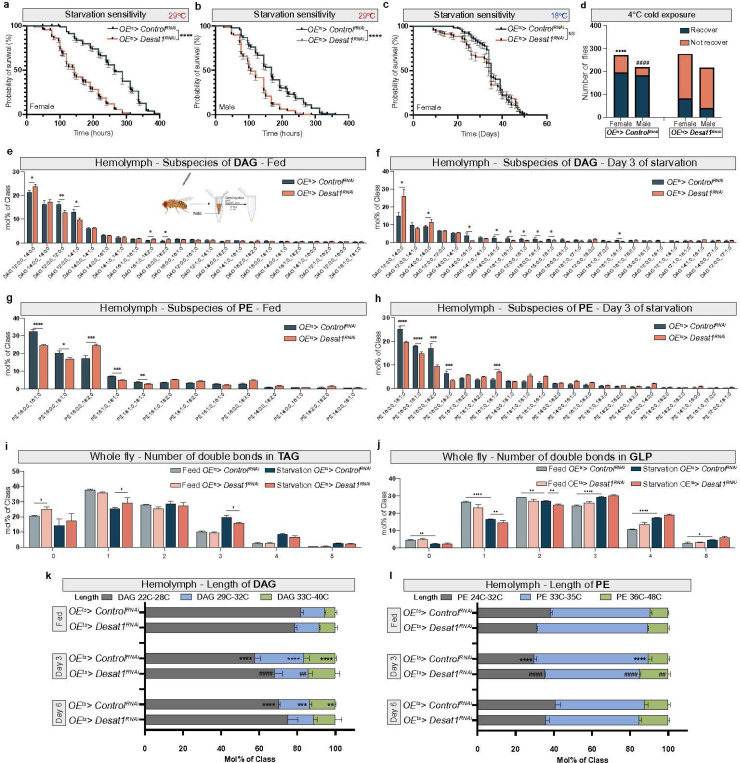
OEts>Desat1RNAi flies exhibit altered lipid profiles and higher sensitivity to starvation and cold stress a,b, OEts>Desat1RNAi adult flies show higher starvation sensitivity at 29°C compared with OEts> ControlRNAi flies in both male and female, n=270 in female flies and n=238 in male flies. P value was calculated using Log-rank (Mantel-Cox) test. c, OEts>Desat1RNAi adult female flies show no different in lifetime during starvation compared with control group at 18°C, n=113, P value was calculated using Log-rank (Mantel-Cox) test. d, Both OEts>Desat1RNAi adult female and male flies show a better recovery from cold exposure. 24 hours cold exposure for male adult flies and 48 hours for female flies, n≈217 for male, n≈270 for female. P value was calculated using Chi-square, ****P<0.0001: female OEts>Desat1RNAi vs. OEts>ControlRNAi flies, ####P<0.0001: male OEts> Desat1RNAi vs. OEts>ControlRNAi flies. e-h, Subspecies lipid analysis (diacylglycerol (DAG) and phosphatidylethanolamine (PE)) of hemolymph samples between Desat1 KD and control groups in fed or starvation conditions, n=3. Statistical tests: multiple unpaired t tests. i,j, The number of double bonds in triacylglycerol (TAG) and GLP (glycerophospholipids) from flies samples in fed or starvation conditions, n=3. Statistical tests: two-way ANOVA with Tukey’s multiple comparisons test. k,l, Changes in the percentage of different chain lengths of DAG and PE in hemolymph during starvation, n=3. Statistical tests: two-way ANOVA with Tukey’s multiple comparisons test. #: OEts>Desat1RNAi flies at day 3 of starvation vs. OEts>ControlRNAi flies day 3 of starvation, *: OEts>ControlRNAi flies at day 3 or day 6 of starvation vs. OEts>ControlRNAi flies at feed condition. *, P<0.05; **, P<0.01; ***, P<0.001; ****, P<0.0001. ##, P<0.01; ####, P<0.0001. Error bars indicate standard deviation.

In this study, we established a modified starvation medium for *Drosophila* which is based on the omission of all calories in holidic medium (besides 10kcal/L stemming from the acetic acid to match the pH of natural food sources)^19^. Compared to the commonly used phosphate-buffered saline (PBS)-based starvation media, the medium contains a number of specific ions and metals in a 2% agar medium (see method). As demonstrated in Fig. 1a, a comparative analysis of different starvation protocols revealed that flies with our ion-enriched starvation medium exhibited a significantly extended survival, allowing us to detect metabolic changes over a time period of up to 10 days. Survival extension was more pronounced in females (Fig. 2a,b) and was largely independent of the calories derived from acetic acid supplementation (Extended Data Fig. 1a). We performed lipidomics on whole fly and hemolymph samples at *ad libitum* fed conditions as well as on different days of nutrient restriction (NR). In the whole fly samples (female), TAG levels exhibited a modest elevation at the beginning of NR, followed by a decline in the subsequent stages (Extended Data Fig. 1b). Conversely, levels of PE, phosphatidylcholine (PC) and phosphatidylinositol (PI) increased markedly throughout the starvation period (Extended Data Fig. 1b). We further observed an overall increase in the number of double bonds and length of acyl chains within several lipid classes in both whole-fly and hemolymph samples (Fig.1b-e and Extended Data Fig. 1d-k). In the hemolymph, the biggest double bond increase was found for DAG and PE. Hence, an overall increase in the number of double bonds and lipid chain length in circulating lipoproteins represents the main change in lipid composition during NR.

### Oenocytes control circulating lipid and global TAG levels during starvation in a Desat1-dependent manner

A remarkable hepatocyte-like feature of oenocytes is starvation-induced steatosis, which can be seen both in larval and adult stages (Fig. 1f and g). As Desat1 is highly expressed in these specialized cells^4,10^, we hypothesized that the lipid alterations we observed during starvation may be related to Desat1-dependent lipid storage and release by oenocytes. To study this, we first performed lifespan experiments using flies with and without oenocyte-specific *Desat1* knockdown (KD). As shown in Fig. 2a and b, we indeed found a higher starvation sensitivity in both female and male flies with GAL80-dependent temperature-sensitive expression of *Desat1* RNAi^10^ in oenocytes (hereafter referred to as *OE*^*ts*^*>Desat1*^*RNAi*^) compared to flies expressing an empty vector RNAi construct in oenocytes (*Control*^*RNAi*^). As additional control experiments, we used *GFP* RNAi (Fig. 6f) and maintained the expression of the GAL80 repressor at 18°C (Fig. 2c). Both of these controls did not show any significant differences in starvation survival.

Cold exposure is another stress condition that involves a global shift towards decreased lipid saturation levels to counteract the decreased membrane fluidity, also referred to as homeoviscous adaptation^20,21^. To test whether Desat1 in oenocytes contributes to this global response, we exposed flies to 4°C for 24 hours (male) or 48 hours (female) and then measured the number of flies that are able to recover. *OE*^*ts*^*> Control*^*RNAi*^ flies showed a greater resistance to 4°C exposures compared with *OE*^*ts*^*> Desat1*^*RNAi*^ flies (Fig. 2d), providing further support that oenocyte Desat1 is important for systemic lipid metabolism in adaptation to starvation and cold stress.

**Figure 3:**
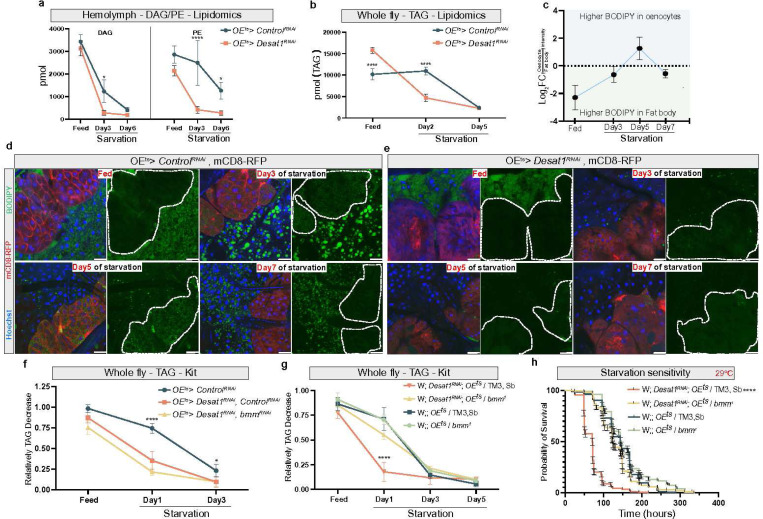
OEts > Desat1RNAi adult flies exhibit a faster lipid consumption in the fat body during starvation a, the amount of DAG and PE at different days of starvation in hemolymph between OEts> Desat1RNAi adult female flies and OEts>ControlRNAi adult female flies measured by mass spectrometry, n=3. Statistical tests: two-way ANOVA with Tukey’s multiple comparisons test. b, The amount of TAG at different days of starvation in whole fly sample between OEts>Desat1RNAi and OEts>ControlRNAi adult female flies measured by lipidomics, n=3. Statistical tests: two-way ANOVA with Tukey’s multiple comparisons test. c, the BODIPY intensity in oenocyte or surround fat body during different days of starvation. d,e, LD staining (green) at different days of starvation. mCD8-RFP (red) was overexpressed in oenocytes to mark the the oenocytes (dashed line). Scale bars: 20 μm. f, TAG level at different days of starvation in control, Desat1 KD and double KD of Desat1 and bmm specifically in oenocytes measured by TAG kit, n=4. Statistical tests: two-way ANOVA with Tukey’s multiple comparisons test. *: OEts>ControlRNAi vs. OEts>Desat1RNAi or OEts> Desat1RNAi, bmmRNAi adult female flies. g, TAG level at different days of starvation measured by TAG kit, n=3. Statistical tests: two-way ANOVA with Tukey’s multiple comparisons test. *: Desat1 KD group vs. all other groups. h, Starvation sensitivity assay between control flies, Desat1 KD flies and Desat1 KD flies carrying one alle of bmm loss-of-function mutation, n=180. P value was calculated using Log-rank (Mantel Cox) test. *, P<0.05; ****, P<0.0001. Error bars indicate standard deviation.

To directly study the impact of oenocyte Desat1 on lipid compositions, we measured the global and hemolymph lipidomes of *OE*^*ts*^*>Desat1*^*RNAi*^ and control flies under normal and NR conditions. As shown in Fig. 2e-h, the saturation level of several major lipids in the hemolymph from *OE*^*ts*^*>Desat1*^*RNAi*^, particularly in DAG and PE, were elevated compared to the control group in both fed and starved conditions. By contrast, we observed only modest changes in the number of double bonds in TAGs and all glycerophospholipids (GPLs) in whole fly samples (Fig. 2i,j). However, the differences of all lipids in both whole fly and hemolymph between the OE^ts^*>Control*^*RNAi*^ and *OE*^*ts*^*> Desat1*^*RNAi*^ groups appeared more pronounced in NR compared to fed conditions (Extended Data Fig. 2a,b). In particular, the starvation-induced increase of acyl chain length of most hemolymph lipids was suppressed in the *OE*^*ts*^*>Desat1*^*RNAi*^ group (Fig. 2k,l), altogether indicating that Desat1 function in oenocytes controls both saturation level and length of the circulating lipidome. We further noticed that lipoproteins levels, as reflected by hemolymph DAG and PE levels, dropped during starvation, and that this drop was much faster in the *OE*^*ts*^*>Desat1*^*RNA*i^ group (Fig. 3a). This was accompanied by a decrease in the global TAG levels in the *OE*^*ts*^*> Desat1*^*RNAi*^ group. Particularly, on the second day of starvation, there was a dramatic decrease in TAG levels compared with the *OE*^*ts*^*>Control*^*RNAi*^ group (Fig. 3b).

In summary, these finding suggest that oenocytes are capable of processing and releasing lipids into the hemolymph as well as controlling global TAG levels in a Desat1-dependent manner during NR. Considering TAG’s role as a primary energy source during starvation, this rapid lipid utilization likely explains the higher starvation sensitivity of *OE*^*ts*^*>Desat1*^*RNAi*^ flies.

### Oenocytes regulate FB lipid storage in a non-autonomous manner

To assess the TAG dynamics during the NR period, we performed BODIPY staining at day 3, 5 and 7 of NR. In *OE*^*ts*^*>Control*^*RNAi*^ flies, LD accumulation in oenocytes (marked with co-expression of CD8-RFP) was increased during the early and middle phases of starvation, with a noticeable decrease in the later stages. Interestingly, the adjacent FB began to exhibit to accumulate small LDs beyond day 5, suggesting a bidirectional lipid exchange between oenocytes and FB (Fig. 3c,d). In *OE*^*ts*^*> Desat1*^*RNAi*^ flies, we detected significantly reduced LD formation at all stages of starvation (Fig. 3e), in line with previous studies on the role of SCDs in TAG synthesis^12,13^. Similarly, when silencing *Desat1* only in the FB, a strong suppression of LD formation in the FB as well as global TAG content was observed. This phenotype was coupled with a strong developmental arrest at larval stages (Extended Data Fig. 3 a-f).

An important additional finding in *OE*^*ts*^*> Desat1*^*RNAi*^ flies was the non-autonomous effect on the FB. The surrounding FB exhibited a sharp decline of BODIPY staining at the onset of starvation, suggesting an accelerated lipolysis in the FB compared to the control (Fig. 3e). Therefore, we hypothesized that oenocytes might regulate global TAG levels by controlling TAG levels in the neighboring FB. To rule out that global TAG depletion is after all the result of enhanced lipolysis in the oenocytes, we first co-silenced *Desat1* and *Brummer* (*bmm*), the main neutral TAG lipase (ATGL in mammals), in oenocytes and monitored TAG levels over the course of starvation. Intriguingly, akin to the *OE*^*ts*^*> Desat1*^*RNAi*^ group, the whole-body TAG levels in the *Desat1/bmm* double KD group still exhibited a significant drop by day 2 of starvation (Fig. 3f), despite the accumulation of LDs within the oenocytes (Extended Data Fig. 4 a,b). For global lipolysis reduction, we next removed one copy of *bmm* in *OE*^*ts*^*> Desat1*^*RNAi*^ fly. Remarkably, this genetic strategy fully rescued the increased starvation sensitivity of *OE*^*ts*^*> Desat1*^*RNAi*^ flies. Furthermore, global TAG consumption in *OE*^*ts*^*> Desat1*^*RNAi*^ animals was suppressed in the heterozygous *bmm*^1^ background (Fig. 3g,h). Together with the strong LD decline in the FB of *OE*^*ts*^*> Desat1*^*RNAi*^ animals, this indicates that oenocytes non-autonomously promote lipid storage in the FB, presumably by suppressing lipolytic activity.

### Desat1 deficiency causes an accumulation of lipoproteins and actin on the oenocyte surface

**Figure 4:**
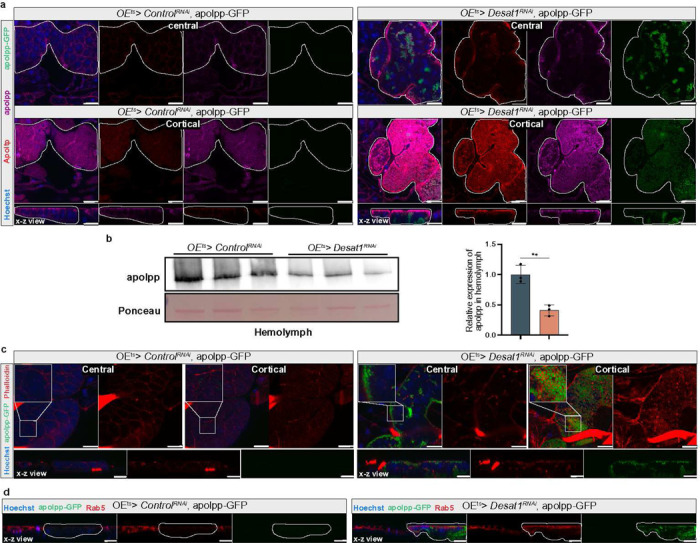
OEts > Desat1RNAi flies causes lipoprotein sequestering in the oenocyte cortex a, Representative immunofluorescence images of lipoproteins in central or cortical sections of oenocytes with or without Desat1 KD. Oenocytes with Desat1 KD exhibit a strong lipoprotein accumulation in the cortex region. X-z view displayed a vertical cross-section along the X-axis and dashed lines showed the oenocytes. Note that the gain in the Desat1 KD group (right panel) was decreased by around 40% to avoid the saturation of image. n>4, scale bars: 20μm. b, Western blot analysis of apolpp in hemolymph from control and Desat1 KD group and quantification of hemolymph apolpp level in hemolymph. Only one band was seen after Ponceau staining, and this was used for normalization. n=3, statistical tests: unpaired t test. c, Representative immunofluorescence images of phalloidin (red) and endogenous apolpp-GFP (green) in central or cortical sections of of oenocytes with or without Desat1 KD. n=3, scale bars: 20 μm. d, X-z view of Rab5 staining and apolpp-GFP with and without Desat1 KD. Dashed lines mark the oenocytes, n=3, scale bars: 20 μm

To further explore the mechanisms by which oenocytes influence hemolymph lipid composition and systemic lipid metabolism, we focused on lipoproteins. For lipoprotein visualization, we used a transgenic line in which apolpp is endogenously tagged with a superfolder green fluorescent protein (sfGFP) at the C-terminus, along with apolpp and Apoltp antibodies for immunofluorescence studies. Intriguingly, a pronounced accumulation of both apolpp and Apoltp was detected on the surface of oenocytes in the *OE*^*ts*^*> Desat1*^*RNAi*^ group, as shown in Fig. 4a. The same was true for the sfGFP signal, although sfGFP and antibody staining in the Desat1 KD group did not fully overlap (Fig. 4a), suggesting potential issues with antibody penetration or partial cleavage of the sfGFP tag. We also performed Western blot analysis using hemolymph samples to assess the levels of lipoproteins in the *OE*^*ts*^*> Desat1*^*RNAi*^ group (Fig. 4b). Consistent with the lipidomics results, there was a marked decrease in apolpp levels in the hemolymph of the *OE*^*ts*^*> Desat1*^*RNAi*^ group (Fig. 4b), suggesting that lipoproteins indeed were sequestered by the oenocytes.

To rule out the possibility that the lipoproteins were after all synthesized in oenocytes, we silenced *apolpp* in oenocytes. As shown in Extended Data Fig. 5a, the apolpp accumulation on the surface of oenocytes in the double KD of *Desat1* and *apolpp* was comparable to the one seen with the single *Desat1* KD. In addition, silencing of *apolpp* in oenocytes did not cause any sensitivity to starvation, altogether suggesting that lipoproteins trapped on the surface of *Desat1*-deficient oenocytes do not originate from oenocytes and most likely are FB-derived.

Imaging with higher magnification revealed that apolpp-GFP accumulated in vesicles close to the cell surface (Fig. 4c). Accordingly, the surface marker CD8-RFP showed a more patchy distribution as well as a more restricted diffusion on the surface of *Desat1* KD oenocytes, as determined by FRAP (fluorescence recovery after photobleaching) experiments (Extended Data Fig. 6). Co-staining with phalloidin and an antibody against the early endosomal marker Rab5 showed that both actin filaments and Rab5 were markedly increased compared to the control and appeared to encapsulate the cortical apolpp vesicles (Fig. 4c,d). Moreover, *Rab5* KD led to a similar accumulation of apolpp and actin (Extended Data Fig. 7), suggesting that impaired endocytosis of lipoproteins might be linked with the actin accumulation.

**Figure 5:**
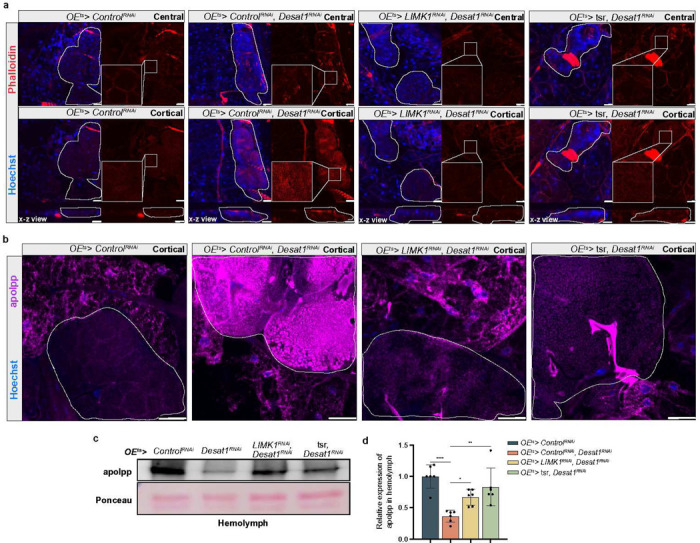
Lowering of F-actin can rescue the sequestration of lipoproteins in oenocytes with Desat1 KD a, Representative immunofluorescence images of phalloidin (red) in central or cortical sections of oenocytes. Actin depolymerization via knocking down Limk1 or overexpressing Tsr reduces cortical actin accumulation. n=4, scale bars: 20 μm. b, Representative immunofluorescence images of apoLpp in central or cortical sections of oenocytes. Dashed lines mark the oenocytes, n=3, scale bars: 20 μm. c, Western blot analysis of apolpp in the hemolymph. OEts>Desat1RNAi exhibit a reduced level of apolpp in the hemolymph compared with OEts>ControlRNAi, which can be rescued by actin depolymerization. Only one band was seen after Ponceau staining, and this was used for normalization. d, Quantification of apolpp in hemolymph, n=6. Statistical tests: one-way ANOVA. *, P<0.05; **, P<0.01; ****, P<0.0001

Based on these findings, we reasoned that a decrease in actin accumulation might be able to release lipoproteins from the oenocytes into the hemolymph. Therefore, we attempted to depolymerize the excessive F-actin by overexpressing *Tsr (twinstar)* or knocking down *Limk1* (LIM domain kinase 1)^22,23,24^. Remarkably, both treatments not only led to a suppression of the actin accumulation (Fig. 5a) but also to a strong decrease of the apolpp accumulation on the oenocyte surface (Fig. 5b) as well as a normalization of lipoprotein levels in the hemolymph (Fig.5 c,d).

Altogether, these results demonstrate that impaired lipoprotein internalization is associated with a rigid meshwork of actin and endosomal vesicles in the oenocyte cortex and that lowering F-actin in oenocytes can normalize lipoproteins circulating hemolymph levels in *OE*^*ts*^*>Desat1*^*RNAi*^ animals.

### Single nucleus RNA-seq identifies candidate proteins secreted from oenocytes during nutrient restriction

**Figure 6:**
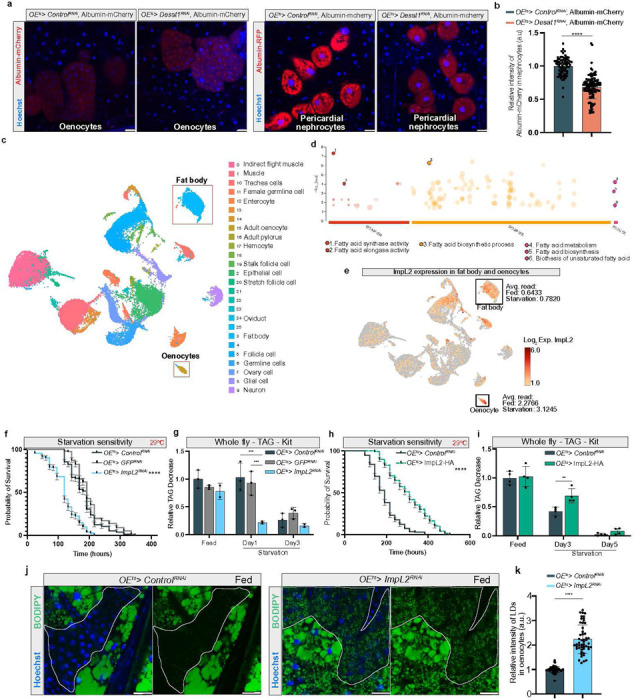
Single-nucleus RNA-seq identifies ImpL2 as important factor during starvation a, Representative immunofluorescence images of albumin-mCherry in oenocytes or pericardial nephrocytes. Albumin-mCherry secreted by oenocytes into the hemolymph is taken up by pericardial nephrocytes. n=4. b, Quantification of relative intensity of albumin-mCherry in pericardial nephrocytes, each data point represents the relative intensity of albumin-mCherry in a pericardial nephrocyte. scale bars: 20 μm. Statistical tests: unpaired t test, ****, P<0.0001. c, A uniform manifold approximation and projection (UMAP) plot. Each color and dot in the plot represented a cluster and a single nucleus, respectively. d, Gene enrichment analysis of these significant different genes (day 2 of starvation vs. fed) was performed demonstrating that many genes were involved in lipid metabolism. e, ImpL2 expression pattern shows a high expression level of impL2 in oenocytes. f, Starvation assay in flies with oenocytes specific ImpL2 knockdown at 29°C. Both OEts>ControlRNAi and OEts>GFPRNAi flies were represented control groups. n=151, P value was calculated using Log rank (Mantel-Cox) test. ****, P<0.0001, OEts>ImpL2RNAi vs. OEts>ControlRNAi or OEts>GFPRNAi. g, TAG levels in whole fly sample were measured at different time point of starvation. n=3, statistical tests: two-way ANOVA with Tukey’s multiple comparisons test. ***, P<0.001. h, Starvation assay in flies with oenocytes specific ImpL2-HA overexpression at 29°C. n=200, P value was calculated using Log-rank (Mantel-Cox) test. ****, P<0.0001. i, TAG level in whole fly sample were measured by TAG kit at different time point of starvation. n=4, statistical tests: multiple unpaired t tests. **, P<0.01. j, LDs (green) during fed condition in oenocytes with ImpL2 knockdown, n=3. k, Quantification of relative intensity of LDs in oenocytes, each data point represented the relative intensity of BODIPY signal in a cluster of oenocytes. Dashed lines mark the oenocytes. Statistical tests: multiple unpaired t tests, ****, P<0.0001. Error bars indicate standard deviation

To test how the alterations of the oenocyte cortex in *Desat1* KD cells might impair exocytosis, we overexpressed albumin-mCherry, whose secretion into the hemolymph can be monitored in pericardial nephrocytes expressing cubilin for albumin uptake. A notable accumulation of albumin-mCherry within the oenocytes was accompanied by a diminished mCherry signal in the pericardial nephrocytes in the *OE*^*ts*^*>Desat1*^*RNAi*^ group compared to the control group (Fig. 6a,b). This result suggested that oenocytes with *Desat1* KD exhibit impaired protein secretion capabilities. To further validate this effect in another cell type, we silenced *Desat1* in the FB and examined the secretion of apolpp. Also in this tissue, we observed a strong accumulation of apolpp within the FB (Extended Data Fig. 8), providing additional evidence that that proper Desat1 function is essential for normal cellular secretion.

To identify secreted factors relevant in the tissue crosstalk during starvation, we utilized full-body single nucleus RNA sequencing (snRNA-seq) to identify genes encoding secreted proteins that are altered during starvation. 90 adult flies (excluding the head) at various stages of starvation (fed state, day 2, and day 5 of starvation) were used for snRNA-seq. 25 Cell clusters were visualized using a uniform manifold approximation and projection (UMAP) plot (Fig. 6c). The genes that changed significantly in oenocytes during starvation compared to the fed condition are presented in a volcano plot in Extended Data Fig. 9a. To further elucidate the metabolic pathways activated in oenocytes during starvation, we performed a gene set enrichment analysis using the online tool PANGEA^25^. The results indicated that genes altered at day 2 of starvation were predominantly enriched in gene ontology (GO) sets related to fatty acid metabolism (Fig. 6d). Our findings therefore support the hypothesis that oenocytes actively engaged in lipid processing during periods of starvation.

### ImpL2 secretion by oenocytes controls starvation responses

Using the algorithm shown in Extended Data Fig. 9b, we then selected 33 genes that were annotated as secreted proteins and either changed during starvation at the mRNA level in oenocytes or were mainly expressed in the oenocytes based on Fly Cell Atlas^26^. From these 33 genes, we selected 8 genes and measured whole-body TAG levels during starvation upon KD in oenocytes (Extended Data Fig. 9c). As flies with oenocyte-specific silencing of *ImpL2* showed the most significant TAG downregulation at day 3 of starvation, we focused on this gene. The glycoprotein ImpL2, which is an inhibitor of several dILPs^27^, was mainly expressed in oenocytes as previously shown^28^, and its expression in oenocytes increased during starvation (Fig. 6e and Extended Data Fig. 9d^29^). Using immunoblotting, we observed a significant reduction of ImpL2 protein in the hemolymph of *OE*^*ts*^*> ImpL2*^*RNAi*^ flies (Extended Data Fig. 9e), confirming that oenocytes are indeed an important source of hemolymph ImpL2. Additionally, an increase of hemolymph ImpL2 protein could be found during starvation compared to fed conditions (Extended Data Fig. 9f,g).

Next, we tested the role of ImpL2 in our starvation assay using oenocyte-specific ImpL2 KD and ImpL2-HA overexpression. We found that the *ImpL2* KD group showed a significantly higher starvation sensitivity (Fig. 6f), whereas flies overexpressing ImpL2 in oenocytes lived significantly longer and showed higher TAG levels than control flies (Fig. 6h,i). Interestingly, oenocytes with *ImpL2* KD were able to induce an accumulation of LDs even in fed conditions (Fig. 6j,k), suggesting that lack of ImpL2 secretion could lead to a starvation-like condition with stimulation of FB lipolysis.

### Defective ImpL2 secretion in Desat1 deficiency can be rescued by cortical actin depolymerization

**Figure 7:**
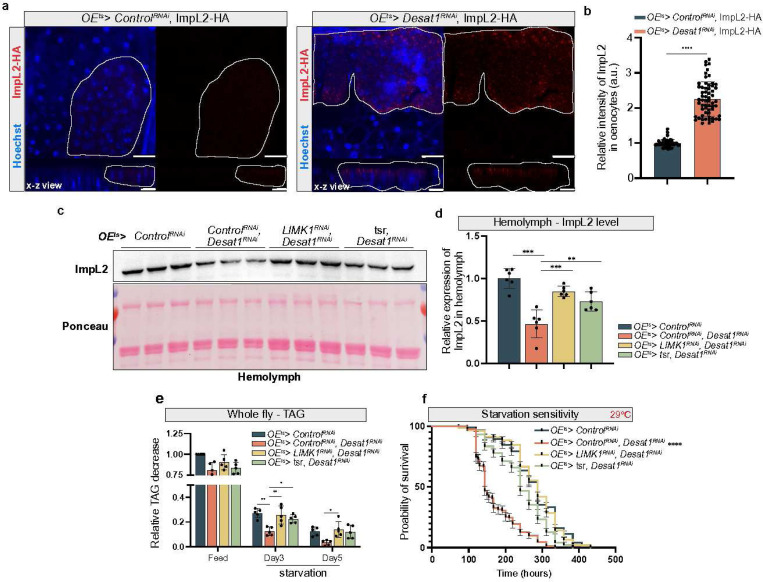
Lowering F-actin rescues the impaired secretion of ImpL2 from oenocytes leading to better starvation resistance a, ImpL2-HA overexpression in oenocytes with or without Desat1 deficiency. ImpL2-HA (red) accumulates in oenocytes in the Desat1 KD group. X-z view shows cortical accumulation in oenocytes (marked by dashed line), n>4, scale bars: 20 μm. b, Quantification of relative intensity of ImpL2-HA in oenocytes, each data point represented the relative intensity of ImpL2-HA signal in a cluster of oenocytes. Statistical tests: multiple unpaired t tests. c, Western blot analysis of ImpL2 in the hemolymph. OEts>Desat1RNAi exhibited a reduced level of ImpL2 in the hemolymph, which can be rescued by actin depolymerization, n=5. d, Quantification of ImpL2 level in hemolymph. Statistical tests: one-way ANOVA, **, P<0.01; ***, P<0.001. e, TAG levels at different days of starvation in whole fly sample measured by TAG kit, n=5. Statistical tests: one-way ANOVA. **, P<0.01; ***, P<0.001. f, starvation assay at 29℃. n=150, P value was calculated using Log rank (Mantel-Cox) test. ****, P<0.0001.

Given the role of Desat1 in protein secretion, we next tested whether secretion of ImpL2 was affected in *OE*^*ts>*^*Desat1*^*RNAi*^ flies. For this, we overexpressed *ImpL2-HA* in oenocytes and conducted immunofluorescence using an HA antibody. Indeed, we found an increase of ImpL2-positive vesicles in cortical regions of oenocytes in *OE*^*ts>*^*Desat1*^*RNAi*^ compared to *OE*^*ts>*^*Control*^*RNAi*^ (Fig. 7a,b). As with the lipoproteins, we tested the effect of depolymerizing the excessive actin by overexpressing *tsr* or silencing *LIMK1*. Also here, these treatments led to a normalization of the ImpL2 levels in the hemolymph of *OE*^*ts>*^*Desat1*^*RNAi*^ animals (Fig. 7c, d). Importantly, *OE*^*ts>*^*Desat1*^*RNAi*^ flies with either *tsr* OE or *LIMK1* KD exhibited longer survival times and higher TAG level compared to flies with *OE*^*ts>*^*Desat1*^*RNAi*^ flies (Fig. 7e, f).

Together, these results indicate that cortical actin depolymerization is able to rescue the defective secretion in *Desat1* KD oenocytes, restoring proper ImpL2 secretion, which in turn suppresses systemic lipolysis and enhances survival during periods of starvation.

## Discussion

**Figure 8:**
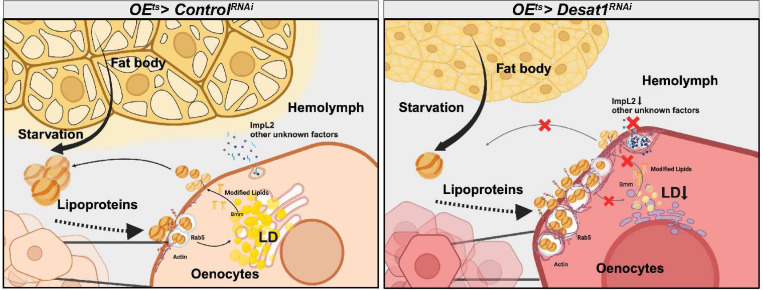
Overview of the role of oenocytes in systemic metabolism during starvation and the impairment by Desat1 deficiency

Our study focuses on the role of oenocytes in regulating lipid dynamics in the hemolymph and the lipid storage functions of the FB during prolonged NR (Fig. 8). Specifically, we show 1) that prolonged NR leads to changes in the circulating lipidome and to storage and release of lipids by oenocytes; 2) that oenocyte expression of *Desat1* is essential for driving the lipidomic changes and for promoting lipid storage in the neighboring FB; 3) that *Desat1* KD leads to lipoprotein sequestering on oenocyte surface and cortical actin accumulation; 4) that *Desat1* KD leads to impaired protein secretion, including ImpL2; 5) *ImpL2* controls survival resistance by controlling FB lipolysis; and 6) that cortical actin depolymerization rescues lipoprotein sequestration and ImpL2 secretion defects, promoting FB lipid storage and starvation resistance.

As part of the starvation response, lipid dynamics are significantly altered during starvation, which is crucial for survival. Our results generally suggest that TAG storage in the FB is a central determinant of survival during fasting, which is consistent with previous reports^14,15^. However, as flies survived up to 10 days with our starvation medium, we were able to obtain additional insights into starvation-dependent lipid dynamics and tissue crosstalk. Over the course of a 7-day starvation period, TAG storage in FB and oenocytes was found to behave in an antiparallel fashion: while the first three days are dominated by massive FB lipolysis and oenocyte lipid uptake and storage, a reversal of functions can be observed thereafter. One consequence of the lipid exchange between these two tissues is that also the circulating lipidome is changing: we observed an increase in both the unsaturation level and carbon chain length of lipids in the *Drosophila* hemolymph during starvation, a finding that aligns with those from the human studies^1^.

Why could a shift to unsaturated lipids be important during starvation? First, specific modified FAs released from oenocytes may function as lipokines, regulating metabolic homeostasis between distant organs. For example, Cao et al. reported on a lipid-mediated endocrine network where adipose tissue utilizes a monounsaturated fatty acid, C16:1n7-palmitoleate, as a lipokine in mice^30^. This suggests that similar mechanisms could be at play in other species, where modified fatty acids released by specific cells, such as oenocytes, act as signaling molecules to orchestrate systemic metabolic responses and maintain energy balance across various tissues^30^. Second, unsaturated lipids generally enhance membrane fluidity, which could be essential for maintaining cell functionality under stress. Accordingly, an important requirement for Desat1 was previously demonstrated for the autophagy pathway in *Drosophila*^31^. And third, as proposed in this study, a more unsaturated circulating lipidome could also lead to more TAG storage in the FB. In line with the view that diglyceride acyltransferase 1 (DGAT1), the rate-limiting enzyme in TAG formation, prefers monounsaturated fatty acids over saturated ones^12^, we could previously show that treatment with a SCD inhibitor leads to a significant reduction in LD formation in mouse kidney cells^13^. Here, we observe that *Desat1* KD in FB causes a dramatic depletion of this tissue, while oenocytes without *Desat1* completely lack LDs. However, beyond these cell-autonomous effects, TAG storage in oenocytes was also shown to be a prerequisite for storage in the FB. Therefore, we suggest that Desat1-dependent cycling of lipids through the oenocyte TAG compartment will lead to an enrichment of unsaturated lipids in the circulation, promoting their storage in the FB over time.

The question how the exchange of lipids between FB and oenocytes is carried out led us to take a closer look at the interaction between apolpp and Apoltp with the oenocytes. Historically, research into insect lipoproteins has suggested that these particles load and unload various lipids at the cell surface and function as reusable shuttles within the organism, facilitating the transport and distribution of lipids across different tissues^32,33,34^. Later, also endocytic mechanisms were found to be involved in the lipid exchange, at least in some tissues^18^. An intriguing example is the prothoracic gland where the KD of *Rab5* not only led to an accumulation of apolpp on the cell surface but also to impaired secretory functions, suggesting that in these cells the control of endo- and exocytosis is coupled via a lipid-dependent mechanism^35^. Our data suggest that oenocytes internalize FB-derived lipoproteins in a Rab5-dependent manner before releasing them again into the circulation. Upon *Desat1* KD, this release was impaired, with apolpp and Apoltp exhibiting a dramatic vesicular accumulation close to the surface. This accumulation correlated with impaired endocytosis and an increased cortical actin polymerization ^36,18^, which most likely was the reason why actin depolymerization via cofilin/tsr overexpression or *LIMK* KD was able to improve the release of lipoproteins into the hemolymph as well as the overall survival of starved *OE*^*ts>*^*Desat1*^*RNAi*^ animals.

Beyond lipoproteins, we further identified ImpL2 as a factor that is upregulated in oenocytes during NR and whose overexpression in oenocytes is sufficient to cause higher global TAG levels and starvation resistance. Importantly, upon *Desat1* silencing, hemolymph ImpL2 levels dropped and overexpressed ImpL2 accumulated in the periphery of oenocytes, suggesting that an impaired release of ImpL2 in *OE*^*ts*^*>Desat1* KD animals is at least partially responsible for the starvation sensitivity of these animals. While also here actin depolymerization led to an increased ImpL2 release, it is unclear whether the cortex alterations in Desat1-deficient oenocytes or other actin filament functions along the secretory pathway are responsible the secretion defect of ImpL2. In islet cells under low glucose conditions, for example, treatment with an actin depolymerization agent can enhance insulin secretion by up to 20 times, highlighting the impact of cytoskeletal dynamics on cellular secretion mechanisms^22^.

A very important finding of our study is that the KD of *ImpL2* in oenocytes can induce a starvation-like phenotype with oenocyte steatosis even under normal feeding conditions (Fig. 6j,k). At first glance, it appears counter-intuitive that an inhibitor of insulin promotes starvation resistance given that TAG storage is usually promoted by insulin^37^. On the other hand, insulin signaling also promotes anabolic energy-consuming processes like cell growth, which could be detrimental during fasting and aging^38,39^. Accordingly, some dILPs, such as dILP6 and dILP7, increase during starvation in *Drosophila*^40^, and flies with genetic ablation of dILP-producing IPCs show increased TAG levels and higher resistance of starvation^41^. Likewise, flies with mutations in *chico*, a key component of the insulin pathway, exhibit increased lipid levels despite a reduced body size^42^. Furthermore, a previous study could also show that larvae with a loss-of-function mutation in *ImpL2* exhibit increased sensitivity to NR conditions^43^. It is therefore conceivable that insulin signaling needs to be tightly controlled during fasting, and that the secretion of ImpL2 from oenocytes contributes to this balance. However, it is also possible that ImpL2 acts independently of insulin signaling as it does in the regulation of glycogen stores following starvation^44^.

Overall, our study contributes to the understanding of lipid metabolism during the starvation response. By manipulating Desat1 function, we demonstrate a key role of oenocytes in modifying circulating lipid profiles as well as FB function. As medical implication, the cell-autonomous and non-cell-autonomous effects observed upon *Desat1* KD need to be considered when testing SCD inhibitors in clinical trials^45,46^. Moreover, serum levels of the ImpL2 homolog in humans, the insulin growth factor binding protein 7 (IGFBP7), have been shown to be positively correlated with insulin resistance, body mass index and the risk of metabolic syndrome^47,48^, while the treatment of mouse and human hepatocytes with IGFBP7 increased LDL and VLDL production^49^. Therefore, ImpL2/IGFBP7 may not only represent an ancient hormone that promotes fat storage in the adipose tissue but also a suitable drug target for the treatment of metabolic syndrome in humans.

## Methods

### *Drosophila* husbandry

Flies were reared at either 25°C or 18°C with 65% humidity under a 12-hour light/dark cycle, fed standard laboratory food. For experiments conducted at 29°C, such as the starvation survival assay, crosses were initially maintained at 18°C until they reached the adult stage. Two-day-old adult flies were then collected and transferred to an environment at 29°C and 65% humidity. These conditions were maintained for one week with a 12-hour light/dark cycle, during which flies were fed either standard or modified starvation food before beginning the experiments.

### Flystocks

Flies used in this study are listed in Supplementary Table 1. For the Desat1 KD we used two independent RNAi lines (V104350KK and v33338GD from VDRC, data shown here were obtained with the GD line). UAS-albumin-mCherry flies were generated by subcloning human albumin from the pGEM-T albumin vector (Sino Biologicals #HG10968-G) first into pmCherry-N1 (Clontech) before subcloning albumin-mCherry further into pUASattB. The construct was injected into flies with the attP landing site at 51C1 by Bestgene, Inc.

### Conditional expression of UAS transgenes

The oenocyte-specific driver, *PromE-Gal*4^50^, was combined with a ubiquitously expressed temperature-sensitive Gal80 (OE^ts^>). To activate the PromE-gal80^ts^ driver, flies were first maintained at 18°C until the adult stage, and two days after eclosion, flies were transferred to 29°C for one week.

### Starvation sensitivity assay in adult flies

To generate age-synchronized adult flies, larvae were raised on standard laboratory food at a low density at 18°C. After eclosion, two-day-old flies were transferred to conditions of 29°C and 65% humidity and allowed to mate for one week. The flies were anesthetized using a low level of CO2, and mated females were sorted at a density of 15 per vial. Flies were transferred to fresh food vials every second day, and mortality was monitored twice daily. The modified starvation food medium was prepared according to the protocol described^19^ with modifications. This medium contains all physiologically relevant ions, including biometals, but lacks energy sources such as sugars, proteins, amino acids, lipids, lipid-related metabolites, nucleic acids, and vitamins (Table 2 and 3).

### Cold exposure experiment

Flies were reared at 18℃ and two-day-old flies were transferred to conditions of 29°C and 65% humidity and allowed to mate for one week. After that, male flies were transferred to a fresh vial (15 flies per vials, around 15 vials for each group) and then kept for 24 hours at 4℃ before transferring them to 25℃ for flies for recovery. After 2 hours, the number of recovered flies were recorded. Female flies were kept for 48 hours at 4℃.

### Immunostaining, lipid droplet labeling and tissue imaging

Dorsal abdominal cuticles containing both FB and oenocytes were dissected in PBS and fixed with 4% paraformaldehyde (PFA) for 25 minutes at room temperature. After fixation, the cuticles were washed for three times and incubated with 0.1% PBST (PBS with 0.1% Triton x-100) for 30min. Then, samples were incubated with a solution of 5% goat serum for 30 min blocking at room temperature before incubating overnight with primary antibodies at 4℃ diluted in 0.1% Tween 20 (guinea pig anti-apolpp^51^, 1:500; rabbit anti-Apoltp, 1:1000 (both from former Eaton lab)^16^; mouse anti-HA, 1:200, (from Santa Cruz sc-7392); rabbit anti-Rab5,1:200 (from Abcam ab31261)). Following this step, the cuticles were washed three times for 10 minutes each and incubated with fluorescent conjugated secondary antibodies (Alexa Fluor 488, Alexa Fluor 555, Alexa Fluor 647 from Invitrogen) at a dilution of 1:200, Alexa Fluor^™^ 555 Phalloidin at dilution of 1:500 and Hoechst at 1:1000 in 0.1% Tween 20 for 2 hours at room temperature. For lipid droplet labeling, BODIPY 493/503 (D3922, Thermo Scientific) was diluted to 2.5ug/ml in PBS and added during the Hoechst staining. Tissues were mounted in Antifade mounting medium (H-1000, Biozol). Images were performed on either a Leica SP5 or Zeiss LSM 780 confocal microscope using a 63x oil-immersion objective. Images were processed using Fiji software.

### Hemolymph extraction and Western blots

To extract hemolymph from adult flies, 30–35 flies were first anesthetized using CO_2_. Each fly was then carefully pierced at the thorax with a tungsten needle and immediately transferred to 0.5 ml Eppendorf tubes that had three holes punctured at the bottom using a 24-gauge needle. These tubes were then placed into a 1.5 ml Eppendorf tube and kept on ice. The samples were centrifuged at 5000 rpm for 5 minutes at 4°C. Following centrifugation, 1 μl of hemolymph was transferred to a new Eppendorf tube and snap-frozen. For Western blot analysis, 1 μl of hemolymph was lysed in RIPA buffer (Thermo Scientific, #89900) supplemented with a protease inhibitor cocktail (Roche, 11697498001) and a phosphatase inhibitor (Roche, #04906845001). The lysate was shaken properly at 4°C for 30 minutes. Subsequently, 4x Laemmli sample buffer (Bio-Rad) and 2% beta-mercaptoethanol were added to the samples, which were then boiled for 5 minutes at 95°C. Lysates were subjected to SDS-PAGE electrophoresis using either 4–15% Mini-PROTEAN^®^ TGX^™^ Precast Protein Gels from Bio-Rad or homemade SDS gels. Proteins were then transferred onto nitrocellulose membranes using the iBlot2 dry blotting system (iBlot^™^ 2 Transfer Stacks, Thermo Fisher). After the transfer, the membrane was stained with Ponceau S for 10 minutes to verify protein transfer and then blocked in Tris-buffered saline with 0.1% Tween-20 (TBST) containing 5% milk at room temperature for 90 minutes. The membrane was incubated with the primary antibody overnight at 4°C (rat anti-apolpp, 1:3000 (from former Eaton lab)^52^; rabbit anti-ImpL2, 1:2000 (from L.Partridge)^53^. Subsequent washes involved three 10-minute TBST rinses before incubation with a horseradish peroxidase (HRP)-conjugated secondary antibody (anti-rat, 1:10000, 31470, Thermo Scientific; anti-Rabbit, 1:10000, SA1–200, Thermo Scientific) in TBST for 90 minutes at room temperature. After four additional 10-minute washes in TBST, protein bands were visualized using SuperSignal West Dura Extended Duration Substrate (34076, Thermo Scientific).

### TAG measurement

We utilized the Triglyceride Assay Kit (TR0100) from Sigma to measure TAG levels. For the assay, three flies constituted one replicate, with three replicates analyzed in total. Lipids were extracted following the kit’s protocol. Each sample, consisting of three flies and 300 μl of 5% NP-40/ddH2O, was added to a 2 ml tube containing a tungsten carbide bead (Qiagen, 69997). The samples were homogenized using a pre-cooled TissueLyser LT for 3–4 cycles, each lasting 4 minutes. Samples were then heated to 90°C in a water bath for 4 minutes, cooled to room temperature, before repeating the heating process once more. After two heating cycles, samples were centrifuged for 2 minutes at maximum speed in a microcentrifuge. The supernatant was diluted fivefold with NP-40/ddH2O before proceeding with the assay. TAG concentrations were measured using a Spark^®^ Multimode Microplate Reader at OD 570 nm and calculated against a standard curve.

### Lipidomics

Mass spectrometry-based lipid analysis was conducted by Lipotype GmbH (Dresden, Germany), using methods described by ^54^. Lipids were extracted following the two-step chloroform/methanol procedure outlined by ^55^. The samples were spiked with an internal lipid standard mixture containing various lipid markers (e.g., CL, Cer, DAG, HexCer, LPA, LPC, LPE, LPG, LPI, LPS, PhA, PC, PE, PG, PI, PS, CE, SM, Sulf, TAG, and Chol). Post-extraction, the organic phase was transferred to an infusion plate and dried using a speed vacuum concentrator. The first-step dry extract was resuspended in 7.5 mM ammonium acetate in a chloroform/methanol/propanol solution (1:2:4, V:V:V), and the second-step dry extract in a 33% ethanol solution of methylamine in chloroform/methanol (0.003:5:1; V:V:V). Liquid handling was performed using a Hamilton Robotics STARlet robotic platform, equipped with anti-droplet control for precise organic solvent pipetting. Samples were analyzed by direct infusion on a QExactive mass spectrometer (Thermo Scientific) equipped with a TriVersa NanoMate ion source (Advion Biosciences). Analyses were conducted in both positive and negative ion modes, with a resolution of Rm/z = 200 = 280,000 for MS and Rm/z = 200 = 17,500 for MSMS, in a single acquisition. MSMS triggers were set by an inclusion list covering the MS mass ranges in 1 Da increments^56^. MS and MSMS data were integrated to monitor various lipid ions: CE, DAG, and TAG as ammonium adducts; PC and PC O- as acetate adducts; and CL, PA, PE, PE O-, PG, PI, and PS as deprotonated anions. MS only was used to track LPA, LPE, LPE O-, LPI, and LPS as deprotonated anions; Cer, HexCer, SM, LPC, and LPC O- as acetate adducts; and cholesterol as an ammonium adduct of an acetylated derivative^57^. Data were processed and analyzed using in-house-developed lipid identification software, LipidXplorer, and managed with an in-house data system. Only lipid identifications with a signal-to-noise ratio >5 and signal intensity at least fivefold higher than corresponding blanks were considered for further analysis. Results were visualized using Prism9 software.

### Single nucleus RNA sequencing

Single nuclei suspensions were prepared following the protocol described in the Fly Cell Atlas^58^. For the preparation, whole-body flies, excluding heads, were flash-frozen in liquid nitrogen and subsequently homogenized in 1 ml of Dounce buffer. The buffer composition included 250 mM sucrose, 10 mM Tris (pH 8.0), 25 mM KCl, 5 mM MgCl_2, 0.1% Triton-X, 0.5% RNasin Plus (Promega, N2615), 1X protease inhibitor (Promega, G652A), and 0.1 mM DTT. The homogenate was then filtered through a 40 μm cell strainer and a 40 μm Flowmi cell strainer (BelArt, H13680–0040) to obtain the suspension. After initial preparation, the samples were centrifuged, washed, and resuspended in 1X PBS containing 0.5% BSA and 0.5% RNasin Plus. The suspension was then filtered through a 40 μm Flowmi cell strainer (BelArt, H13680–0040) immediately before fluorescence-activated cell sorting (FACS). For nuclear staining, DRAQ7^™^ Dye (Invitrogen, D15106) was used. Sorting was performed using a Sony SH800Z Cell Sorter at the Systems Biology Flow Cytometry Facility at Harvard Medical School. Post-sorting, the nuclei were collected and resuspended at a concentration of 700–800 cells/μl in 1X PBS with 0.5% BSA and 0.5% RNasin Plus.

Single-nucleus RNA sequencing (snRNAseq) was performed following the 10X Genomics protocol (Chromium Next GEM Single Cell 3’ v3.1 Rev D). We conducted two reactions for flies in fed and two reactions for flies in starvation condition at each time point (day 2 and day 5). The snRNAseq data were processed using the Cellranger count pipeline 6.1.1 to generate feature-barcode matrices. To normalize data across different samples, the read depths were equalized, and the matrices were aggregated into a single feature-barcode matrix using the Cellranger aggr pipeline. In total, 43,562 cells were profiled, including 20,395 fly cells in fed condition and 15,264 fly cells at Day 2 of staravtion, and 7,903 control fly cells at Day 5 of starvation. Cell clusters and gene expression levels were visualized using the Loupe Browser 6.

### Fluorescence recovery after photobleaching (FRAP)

The FRAP experiment was conducted using a Zeiss 780m confocal microscope equipped with a Plan-APOCHROMAT 63x/1.4 Oil objective. Fresh abdominal tissue was dissected in PBS and placed on a glass slide with a thin layer of Halocarbon 700 oil (H8898, Sigma) spread on it. A 40mm x 60mm coverslip was then carefully positioned over the tissue. After allowing the setup to stabilize for 6–10 minutes, the slide was mounted on the microscope for imaging and the FRAP experiment. The same settings were maintained for all FRAP experiments: Frame size was set at 512×512 pixels with a zoom of 2. Gain settings varied between 600–850, adjusted for each slide to maximize signal without causing overexposure. Time series were recorded at 3-second intervals, with the duration ranging from 2 to 5 minutes, depending on the observed recovery time. The photobleaching region size was set at 30, and the pinhole was adjusted to 1 Airy Unit (AU). Using Zeiss ZEN 2010 software, the following regions of interest (ROI) were extracted: bleach (BL): the main ROI bleached by the laser; background (BG): a region of only noise (outside of any target fluorescence); reference (REF): a region of fluorescence outside of the bleached region. This is used to determine bleaching as a result of repeated image acquisition. The normalized data was calculated: BL_corr2(t) = BL_corr1(t) / REF_corr1(t) = [BL(t) – BG(t)] / [REF(t)-BG(t)] and processed by Prism 9.

## Supplementary Material

Supplement 1

## Figures and Tables

**Table.1:** Standard laboratory *Drosophila* food (per liter, 1% agar)

Name	Per liter
deactivated yeast	18g
soy flour	10g
cornmeal	80g
malt	40g
corn syrup	5%
propionic acid	0.3%
nipagin	0.2%

**Table.2:** Protocol for modified starvation food (per liter)

Name	Per liter
CaCl_2_ (2.5g per 100ml)	0.5 ml
MgSO_4_ (25g per 100ml)	0.5 ml
CuSO_4_(0.25g per 100ml)	0.5 ml
FeSO_4_(2.5g per 100ml)	0.5 ml
MnCl_2_(0.1g per 100ml)	0.5 ml
ZnSO_4_(2.5g per 100ml)	0.5 ml
MoNa_2_O_4_(20g per 100ml)	0.5 ml
Base buffer (10X)	100 ml

**Table.3:** Protocol for 10X Base buffer (per liter)

Base buffer (10x)	Per liter
Acetic acid	30 ml
KH_2_PO_4_	30 g
NaHCO3	10 g
