# Supplementary material for: Desaturase-dependent secretory functions of hepatocyte-like cells control systemic lipid metabolism during starvation in Drosophila
Source: Res Sq. 2024 Dec 11:rs.3.rs-5566817. Preprint. [Version 1] doi: 10.21203/rs.3.rs-5566817/v1 (PMC11661362; doi:10.21203/rs.3.rs-5566817/v1)
Supplement: Supplement 1 [file NIHPPRS5566817v1-supplement-1.pdf]

Supplementary Files

This is a list of supplementary files associated with this preprint. Click to download.

- SupplementaryTable1JY.docx
- ExtendedDatafile.pdf
